#  Intrapersonal predictors of weight bias internalization among elementary school children: a prospective analysis

**DOI:** 10.1186/s12887-020-02264-w

**Published:** 2020-08-29

**Authors:** Michaela Silvia Gmeiner, Petra Warschburger

**Affiliations:** Department of Psychology, University of Potsdam, Karl-Liebknechtstr. 24-25, 14476 Potsdam, Germany

**Keywords:** Weight bias internalization, Self-stigmatization, Weight, Children, Predictors

## Abstract

**Background:**

Weight-related stigmatization is a widespread problem. Particularly the internalization of weight-related stereotypes and prejudices (weight bias internalization, WBI) is related to mental and physical health impairments. To date, little is known about the risk factors of WBI. Previous studies are mainly cross-sectional and based on adult samples. As childhood is a sensitive period for the development of a healthy self-concept, we examined predictors of WBI in children.

**Methods:**

The final sample included 1,463 schoolchildren (6–11 years, 51.7% female) who took part in a prospective study consisting of three measurement waves. The first two waves delivered data on objective weight status and self-reported weight-related teasing, body dissatisfaction, relevance of one’s own figure, self-esteem and depressive symptoms; WBI was measured during the third wave. To examine predictors of WBI, we ran hierarchical regression analyses and exploratory mediation analyses.

**Results:**

Lower parental education level, higher child weight status, female gender, experience of teasing, higher body dissatisfaction, higher figure-relevance, and higher depression scores were found to be predictive for higher WBI scores. Body dissatisfaction (only for girls) and the relevance of one’s own figure (both genders) mediated the association between self-esteem and WBI; no weight-related differences were observed.

**Conclusions:**

Our study offers longitudinal evidence for variables that enable the identification of children who are at risk for WBI. Thus, the findings deliver starting points for interventions aimed at the prevention of adverse health developments that come along with WBI.

## Background

Weight-related stigma is widespread in various areas of life (e.g., interpersonal relationships, health system, education, media). For instance, people with obesity are characterized as lazy, incompetent, sloppy or uncontrolled and potentially face discrimination [1]. This is connected with health-damaging behavior patterns, such as reduced physical activity and disordered eating; long-term consequences include decreased impaired physical (e.g., cardiovascular disease) and psychological health (e.g., impaired self-esteem and body image, depression, anxiety or social isolation) [1–3].

Weight stigma is established in childhood [1]. There is evidence that anti-fat stereotypes already develop from the second year of life onwards [4]. Anti-fat stereotypes might manifest in the experience of stigmatization in social relationships (e.g. being laughed at or excluded from activities of peers). These negative social experiences are risk factors for the emergence of mental problems and establishing a negative self-concept [5]. Since children are more dependent than adults on the social feedback from their environment, children are particularly vulnerable to negative consequences of WBI [1, 3, 6].

According to the self-stigma process that is described for mental illness [7], experiencing weight stigma as part of social norms might result in self-devaluation. This process is labeled as weight bias internalization (WBI) and describes the extent to which people agree with weight-related ascriptions and apply them to themselves [8]. WBI further amplifies the negative consequences of stigmatization and is possibly more important than the mere weight status and experience of stigmatization [2, 9, 10]. For instance, WBI mediates the relationship between weight status and psychosocial problems [11]. Since an early onset of mental problems is associated with increased adverse health outcomes [12], an increased focus on children is needed to prevent these adverse trajectories.

Stigma-related research suggests that the experience of stigma does not necessarily result in its internalization [13]. This prompts the question of which factors further facilitate or impede WBI. Ratcliffe and Ellison [14] have addressed several variables that might enhance vulnerability to WBI. Obviously, higher weight status and experienced weight stigmatization are detrimental factors for WBI in children and adolescents [11, 15–18]. Beyond that, the authors [14] postulate that body dissatisfaction, self-esteem and emotional problems are not only consequences of, but also predisposing factors for WBI. However, prospective studies for children and adolescents are lacking. In cross-sectional studies, more pronounced body dissatisfaction is reported to be associated with higher WBI scores [16, 19], and several studies have found a negative correlation between self-esteem and WBI [11, 18, 20, 21]. Just one study that included only children with overweight reported no significant association between self-esteem and WBI [22]. In addition, there is consistent cross-sectional evidence that WBI is associated with emotional problems [11, 16, 17, 19–21].

Research among adults suggests that sociodemographic variables, such as socioeconomic status, gender and age, might also contribute to WBI [23, 24]. With regard to children, no effects of age on WBI were observed across different age groups [15–19]. Concerning gender, previous evidence is inconsistent. Several studies have observed higher WBI among girls compared to boys in samples with and without overweight [11, 16, 18, 20], whereas other studies reported higher WBI for girls only in the normal-weight subsample [17] or did not find significant gender-related differences among treatment-seeking adolescents [15, 19]. So far, socioeconomic status (SES) has only been considered by one study [18], which found no association with WBI among treatment-seeking adolescents.

To sum up, there is preliminary evidence for psychological correlates of WBI, such as body image, emotional problems and self-esteem, whereas findings on the influence of sociodemographic factors are inconsistent. However, there are some constraints one should take into consideration: First of all, nearly all studies were cross-sectional (except [11]), and therefore only allow associations to be established between different variables but are unsuitable for evaluating causation or temporality. Second, because most evidence stems from validation studies, interactive effects of the variables have not yet been considered. Third, the majority of the research focused on clinical samples with higher weight status, and therefore the results cannot be generalized across different weight groups. This is important, since studies have shown that WBI occurs across different weight groups [16]. Therefore, we aimed to examine the specific contribution of multiple predictors of WBI among primary school children across all weight categories in a prospective study. We expected female gender, lower SES, higher weight status and experienced weight-related teasing in addition to higher body dissatisfaction, lower self-esteem and depressive symptoms to be predictive for children’s WBI over the long term.

## Methods

### Procedure

Data collection was part of a prospective study that investigated intrapersonal developmental risk factors in childhood (PIER-Study, University of Potsdam). Measurements took place in 2012 (T1), 2013 (T2) and 2015 (T3). Families (children and their parents) were recruited in 33 elementary schools representing different socioeconomic backgrounds in Germany (Brandenburg). Parents provided informed and written consent. Participation was voluntary. Measurements with the children (about 50 min each) took place in quiet rooms at their schools or at home. The predictor variables refer to T1 and T2 assessment points; the outcome variable (WBI) was only collected at T3. Children received small presents (buttons or candy) and (book) vouchers as incentives. The local ethics committee approved the study.

### Sample characteristics

The final sample comprised 1,463 children who answered the questionnaire for the main outcome (WBI, 157 cases were excluded due to missing data) and questionnaires at T1 and T2 (40 cases were excluded due to missing data). At T1, children were 6 to 11 years old (*M* = 8.35, *SD* = 0.94), 51.74% of whom were female. According to national reference data [25], 6.08% were categorized as underweight, 80.72% as normal weight and 13.19% as overweight or obese. With respect to educational background, 39.99% of the parents reported a higher education degree, 17.36% reached higher education entrance qualifications and 31.51% reported completion of a secondary school diploma or below; 11.14% did not give information about their education level.

### Materials and measures

#### Sociodemographic and anthropometric data

SES was assessed by parental report of the highest graduation level (reported by *n* = 1,300 parents). Study personnel documented the children’s gender; age was calculated based on birthdates. Study personnel measured the children’s height and weight with calibrated instruments. Based on national reference data, body mass index standard deviation scores (BMI-SDS, z-scores) were calculated and weight groups were classified based on percentiles (> 90th percentile: overweight; > 97th percentile: obesity) [25].

#### Weight bias internalization

The modified Weight Bias Internalization Scale for children (WBIS-C) [16] assesses to what extent children apply weight-related stigma to themselves (e.g., “Because of my weight I don’t deserve having a lot of friends and fun.”). All items were rated on a 4-point Likert scale (*I disagree, I somewhat disagree, I somewhat agree, I agree*), with higher mean values indicating a higher agreement with WBIS-C items. The scale showed satisfying psychometric properties and reliability (α = 0.86), as well as factorial and convergent validity [16]. Cronbach’s alpha in the current sample was α_T3_ = 0.86.

#### Weight-related teasing

Children rated the frequency (*never, sometimes, often*) of weight-related teasing (“Are people mean to you because you are fat?”; 1 item) and weight-related exclusion (“Are you being excluded by other children because you are fat?”; 1 item) on two items taken from the Perception of Teasing Scale [26]. Higher mean scores indicated more frequent experiences of weight-related teasing. Internal reliability was adequate in the current sample (α_T1_ = 0.62, α_T2_ = 0.74).

#### Psychosocial variables

Body dissatisfaction (“Are you satisfied with your figure?”; this item was reversed for analyses) and the relevance of one’s own figure (“Is your figure important to you?”) were assessed by self-constructed items. Items were rated on a 4-point Likert scale (*I disagree, I somewhat disagree, I somewhat agree, I agree*).

Self-esteem was assessed by the subscale “self-esteem” of the KINDL-R, which is a valid and reliable (α = 0.70) self-report questionnaire [27]. The four items (e.g., “I was proud of myself.”) were rated on a 3-point Likert scale (*never, sometimes, often*), with higher scores indicating higher self-esteem. Cronbach`s alpha reached α_T1_ = 0.42 resp. α_T2_ = 0.46.

Depressive symptoms were self-reported by four dichotomous items (*yes/no*, e.g., “Are you sad or gloomy?”). The items are based on the Diagnostic System for Mental Disorders in Childhood and Adolescence (DISYPS-KJ), which is a valid instrument to detect mental disorders in children [28]. Higher sum scores indicated the occurrence of more depressive symptoms. Internal consistency was α_T1_ = 0.49 and α_T2_ = 0.5.

### Analyses

#### Data preparation

Preliminary analyses revealed no consistent violation of assumptions for regression (linearity, normal distribution of residuals, homoscedasticity, independence of the residuals). Only two variables showed slight (BMI-SDS) or notable (relevance of figure) heteroscedasticity. Therefore, we ran bootstrapping (with 2,000 samplings) for the regression analysis in order to prevent biased results [29].

BMI-SDS was revealed to be highly stable over time (*r*_T1T2_ = 0.9, *r*_T2T3_ = 0.87, *r*_T1T3_ = 0.84; *p* < 0.001). Therefore, we replaced missing BMI-SDS data on the individual with their own previous or subsequent BMI-SDS scores. For all remaining variables, missing data with a missing rate of less than 5% were imputed via EM-algorithm [30, 31]. This criterion was fulfilled by all variables except for parental education (missing rate of 11%). Due to randomly missing data, further variables were included in the estimation process. To check for possible bias, all analyses were carried out with and without imputed data.

#### Prediction of WBI at T3

We used a stepwise approach to include relevant variables. In a first step, potential predictors at T2 were correlated with the WBIS-C at T3. Based on the scale level, we applied pointbiserial correlation for gender and polyserial correlation for parental education. All other variables were analyzed using Pearson’s correlation. In a second step, variables showing a significant correlation were entered into a regression analysis to predict the WBIS-C score in the following order: (1) sociodemographic variables (age, gender, parental education, using 3 dummy variables with the highest category (higher education degree) as a reference), (2) BMI-SDS, weight-related teasing and (3) further psychosocial factors (body dissatisfaction, relevance of one's own figure, self-esteem and depressive symptoms).

All analyses were conducted with SPSS version 25. The alpha significance level was set to α < 0.05 for all analyses. Effect sizes (*r*, Cohen’s *d*) were interpreted according to Cohen [32].

## Results

The mean WBIS-C score was 1.62 (*SD* = 0.59). Compared to boys (*M* = 1.56, *SD* = 0.56), girls reported significantly higher WBIS-C scores (*M* = 1.68, *SD* = 0.62; *t*(1461) = 3.89, *p* < 0.001; *d* = 0.2). Among children with overweight or obesity (*M* = 2.16, *SD* = 0.68), WBIS-C scores were higher than among children with underweight or normal weight (*M* = 1.53, *SD* = 0.52; *t*(1461) = -15.39, *p* < 0.001; *d* = -1.15).

Age was not significantly correlated with the WBIS-C (*p* > 0.05). Correlation analyses revealed significant correlations of WBIS-C with gender (*r* = − 0.13, *p* < 0.001, indicating higher WBIS-C scores for girls). Further descriptive data and correlations with WBIS-C scores are presented in Table 1.

**Table 1:** Descriptive data and correlations with WBIS-C scores

	*M* (*SD*)	correlation WBIS-C	
		*r*	*p*
parental education ^a^	3 (2) ^b^	− 0.27 ^c^	< 0.001
T2 BMI-SDS	0.2 (1)	0.43	< 0.001
T2 weight-related teasing ^d^	1.1 (0.32)	0.28	< 0.001
T2 body dissatisfaction ^e^	1.54 (0.75)	0.36	< 0.001
T2 relevance of one’s own figure ^e^	3.07 (0.9)	0.2	< 0.001
T2 self-esteem ^e^	0.6 (0.86)	− 0.05	0.046
T2 depressive symptoms ^f^	2.3 (0.38)	0.24	< 0.001

*Note.* *N* = 1,463; *WBIS-C* weight bias internalization scale, *BMI-SDS *body mass index standard deviation scores (z-scores)

^a^ higher values indicate a higher education level, categories of parental education: 1 = no graduation/graduation from a special-needs school/secondary modern school qualification, 2 = secondary school diploma, 3 = higher education entrance qualification, 4 = higher education degree; ^b^ median and interquartile range; ^c^ polyserial correlation; range of values: ^d^ 1–3;^e^ 1–4;^f^ 0–3

### Prediction of weight bias internalization

The results of the hierarchical regression analysis are presented in Table 2. Each step contributed significantly to the explanation of variance in WBIS. The model including all variables accounted for 31.2% of the variance. Apart from self-esteem, all T2 variables were significant predictors of T3 WBI.

**Table 2:** Prediction of WBI: Hierarchical regression of T2 variables on T3 WBI

**Independent variables**	Coefficients				Model		
	***B*** **[BCa 95% CI]**	**SE** ***B***	**β**	***p***	*R*^2^	Δ *R*^2^	*p*
**Step 1**							
gender ^a^	-0.11 [-0.17; -0.05]	0.03	− 0.1	< 0.001	0.07	0.07	< 0.001
parental education ^b^							
no graduation/graduation of a special-needs school/secondary modern school qualification	0.47 [0.29; 0.64]	0.09	0.13	< 0.001			
secondary school diploma	0.33 [0.26; 0.41]	0.04	0.26	< 0.001			
higher education entrance qualification	0.18 [0.1; 0.25]	0.04	0.13	< 0.001			
higher education degree	Ref						
**Step 2**							
gender^a^	-0.11 [-0.17; -0.06]	0.03	− 0.1	< 0.001	0.24	0.17	< 0.001
parental education ^b^							
no graduation/graduation of a special-needs school/secondary modern school qualification	0.28 [0.12; 0.44]	0.08	0.08	0.001			
secondary school diploma	0.22 [0.15; 0.29]	0.03	0.17	< 0.001			
higher education entrance qualification	0.13 [0.06; 0.19]	0.03	0.09	< 0.001			
higher education degree	Ref						
BMI-SDS	0.21 [0.18; 0.24]	0.01	0.35	< 0.001			
weight-related teasing	0.27 [0.18; 0.36]	0.04	0.15	< 0.001			
**Step 3**							
gender ^a^	0.1 [-0.15; -0.05]	0.03	− 0.08	< 0.001	0.31	0.07	< 0.001
parental education ^b^							
no graduation/graduation of a special-needs school/secondary modern school qualification	0.21 [0.05; 0.36]	0.08	0.06	0.009			
secondary school diploma	0.17 [0.11; 0.23]	0.03	0.13	< 0.001			
higher education entrance qualification	0.09 [0.03; 0.16]	0.03	0.07	0.004			
higher education degree	Ref						
BMI-SDS	0.17 [0.14; 0.2]	0.01	0.29	< 0.001			
weight-related teasing	0.15 [0.06; 0.23]	0.04	0.08	0.001			
body dissatisfaction	0.17 [0.13; 0.2]	0.02	0.21	< 0.001			
relevance of one’s own figure	0.07 [0.05; 0.1]	0.01	0.11	< 0.001			
self-esteem	-0.05 [-0.11; 0.02]	0.03	− 0.03	0.19			
depressive symptoms	0.08 [0.04; 0.11]	0.02	0.11	< 0.001			

*Note.* *B* unstandardized regression coefficient, *BCa 95% CI *95% bias corrected and accelerated confidence interval, *BMI-SDS *body mass index standard deviation score; BCa 95% CI, standard error and *p*>-values are based on 2,000 bootstrap samples; a positive *B* value indicates that this (lower) category predicts higher WBIS-C scores compared to the category of higher education degree

^a^ bivariate coded: 0 = female, 1 = male

^b^ education level was dummy coded (3 dummy variables (categories 1–3) with highest level (category 4) as a reference [Ref])

### Additional exploratory analysis

**Fig. 1:**
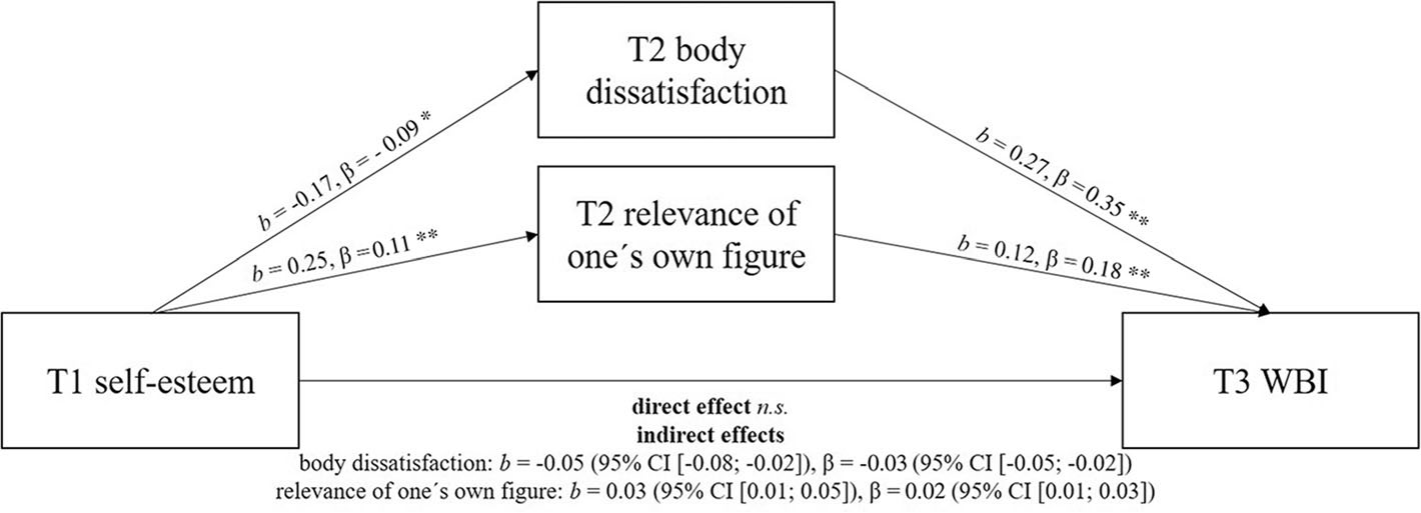
Body dissatisfaction and relevance of one’s own figure as mediators between self-esteem and WBI. 95% CI = 95% confidence interval based on 5,000 bootstrap samples; T1 = first measurement wave, T2 second measurement wave, T3 third measurement wave; WBI = weight bias internalization; ** significant with α ≤ 0.001; * significant with α ≤ 0.01; n.s. = not significant. Consideration of covariates (weight status, parental education, weight-related teasing, depressive symptoms) led to the same result pattern

**Fig. 2:**
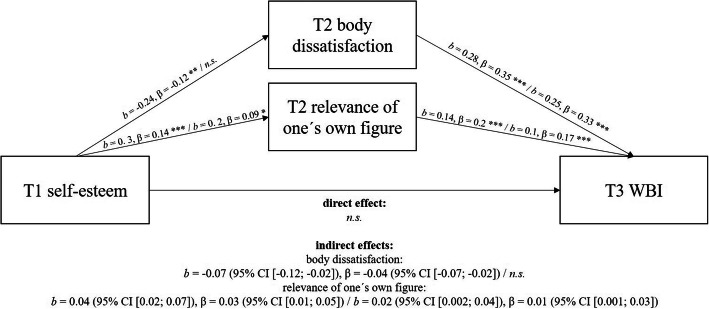
Body dissatisfaction and relevance of one’s own figure as mediators between self-esteem and WBI in girls / boys. Group sizes: 757girls / 706 boys; 95% CI = 95% confidence interval based on 5,000 bootstrap samples; T1 = first measurement wave, T2 second measurement wave, T3 third measurement wave; WBI = weight bias internalization; *** significant with α ≤ 0.001, ** significant with α ≤ 0.01, * significant with α ≤ 0.01; n.s. = not significant. Consideration of covariates (weight status, parental education, weight-related teasing, depressive symptoms) led to the same result pattern

As T2 self-esteem was slightly correlated with T3 WBIS-C but lost significance in the presence of other variables, the possible existence of a mediation process was tested. As explained by another study [33], the association between self-esteem and WBI might be mediated by body image. Hence, additional mediation analysis via the PROCESS macro (model 4) according to Hayes’ recommendations [34] was performed. Since WBI and other psychological variables (as body dissatisfaction) vary by gender and weight group [35, 36], we ran separate models for boys vs. girls and under-/normal-weight group vs. overweight/obese group. We included self-esteem at T1 as the independent variable, and the mediators at T2 and WBI as dependent variables at T3. Figure 1 illustrates the results of the mediation analyses in the overall sample. Both body dissatisfaction and relevance of one’s own figure mediated the relationship between self-esteem and WBIS-C scores. Gender-specific analyses (see Fig. 2) showed that the indirect effect of body dissatisfaction was only significant for girls but not for boys. No weight-related differences emerged.

## Discussion

WBI is not only common but is also associated with detrimental effects on physical and mental health. So far, little is known about who is at greater risk of internalizing weight stigma. Our results suggest that female gender, lower parental education level, higher weight status and experienced weight-related teasing, in addition to self-reported body dissatisfaction, relevance of one’s own figure and depressive symptoms, are predictive for children’s subsequent higher WBI.

Taken together, our prospective observations are mostly in accordance with previous findings from cross-sectional research and expand on the existing body of knowledge by focusing on longer-term effects in a younger age group. First, the results concerning sociodemographic variables highlight that girls are at a higher risk for WBI compared to boys. Beyond that, a higher parental education level goes along with a lower WBI. This is in contrast to a previous study [18] reporting no cross-sectional association between SES and WBI among a group of treatment-seeking adolescents. However, comparison is limited because the two studies differ in terms of operationalization of SES (parental education vs. family affluence via youth report [18]) and the percentage of individuals with a lower SES. Future studies should focus on the mechanisms (e.g., availability of resources, parental behavior) that might explain this association. In accordance with the literature [18, 19, 23], we observed no association between age and WBI in children. This suggests that, among school-aged children, all age groups are equally affected.

We included children across different weight groups and noted, as expected, that experienced weight teasing and higher weight status are relevant for WBI. Previous studies that reported no correlation of WBI with weight status [19, 20] solely referred to participants with overweight/obesity. For individuals within the overweight/obese group, WBI seems to be a problem but the degree of overweight might be of minor importance. Our results concerning negative body image and emotional problems replicated and extended previous results [11, 18–21] by showing that these variables precede WBI and are not just correlates of WBI. Previously, these variables were regarded as consequences of WBI, but it seems important to consider them also as aggravating influences to WBI.

With respect to self-esteem, the present data contradict previous cross-sectional studies showing a negative association with WBI [11, 18, 20, 21]. We observed a slight correlation of T2 self-esteem and T3 WBI, but self-esteem was not predictive in the interplay of other additional psychosocial variables. To interpret this result, several issues have to be taken into account. First, during childhood, self-esteem varies over time [37], which is supported by a low correlation of T1 and T2 self-esteem (*r* = 0.34, *p* < 0.001) in our sample as well. Second, the lack of an additional predictive value while including other psychosocial variables might indicate that self-esteem does not have a direct influence on WBI but rather an indirect one, as shown in a previous study [33]. Our mediation analysis revealed that higher self-esteem was associated with a higher relevance of one’s own figure, which in turn led to higher WBIS-C scores. This pattern contradicts the results of one study [33], which found that lower self-esteem is associated with a higher overvaluation of weight and shape (and in turn higher WBI). Contrary to overvaluation of weight and shape, the mere relevance of one’s own figure does not necessarily mean that individuals overemphasize their own appearance. With respect to body dissatisfaction, we observed the expected mediation pathway between self-esteem and WBI – but only among girls. Research on the role of gender for the relationship between self-esteem and body-dissatisfaction is conflicting. For instance, one study [38] reported a similar gender-specific cross-sectional relationship between self-esteem and body dissatisfaction, while another [39] found no gender-related differences. As these studies refer to adolescents and apply different methodologies (e.g., body dissatisfaction measured by figure drawings versus written items), comparability is limited and the role of gender remains unclear. Future studies should consider gender-specific pathways.

### Strengths, limitations and future implications

The study provides initial prospective data on the emergence of WBI. The interpretation of the results should take into account several limitations of our study: First, our sample mainly refers to individuals with an above-average education level. Strictly speaking, this limits generalization and might underestimate the relevance of a lower parental education level. Second, there are some limitations with respect to operationalization. To reduce subject burden, some constructs were assessed with relatively short scales (depressive symptoms, teasing) or with only one item (body dissatisfaction, relevance of one’s own figure) and with simple rating formats. Certainly, several scales (self-esteem, depressive symptoms) yielded low internal consistency. This is acceptable for diverse psychological constructs, especially for screening purposes [40–42]. In addition, internal consistency was probably influenced by ceiling effects [43]. Low reliability constrains a measurement’s accuracy and limits the magnitude of correlations among variables. Therefore, our results have to be interpreted carefully and should be confirmed with large-scale instruments providing better psychometric properties. Besides, although analyzing Likert-scaled items with parametric procedures is common practice [44, 45], the metric properties of an interval level (as equal distances between scale sections) ultimately cannot be ensured for these variables (for a critical discussion, see e.g. [46]). However, we followed this approach because it enables the application of elaborate analytical methods and is assumed to be a more conservative approach. Furthermore, it has been shown that parametric procedures yield correct results, although the data do not completely fulfill the criteria for interval scales [47]. Furthermore, the teasing items referred to teasing due to overweight and not to weight in general, which might lead to an underestimation of weight-related teasing among children with under- or normal weight. Third, WBI was only assessed at the last measurement wave, preventing us from controlling for its baseline value. Along with this, the design limits conclusions about bidirectional relationships: We can only identify predictors of WBI, but we cannot take into account how WBI might in turn influence self-esteem, body dissatisfaction or emotional problems. Recent studies show that body dissatisfaction, self-esteem and depressive symptoms can also be considered as sequelae of WBI [2]. Taken together, one might postulate a vicious cycle of mutual reinforcement between WBI, self-esteem, depressive symptoms and body dissatisfaction.

The present study also shows several strengths. To our knowledge, this is the first study investigating the risk factors of WBI in a prospective design. Since we focused on schoolchildren, the study is able to add to previous evidence mainly based on adolescent samples. This is of considerable significance, because WBI is connected to reduced mental health outcomes [2], and an early onset of mental problems is associated with increased adverse health outcomes [12]. Further, our analysis is based on a huge sample size including an equal number of girls and boys. As suggested, WBI was assessed with respect to weight in general and not only overweight, therefore allowing conclusions regarding different weight groups [48].

## Conclusions

To prevent WBI, interventions aimed at reducing weight stigma are promising [49] but show only small effects. Therefore, intervention and prevention efforts also might include intrapersonal variables to strengthen those who are at risk.

This study delivers starting points for addressing high-risk populations in selective prevention programs. In order to impede adverse health outcomes of WBI, prevention should be started as early as possible (as age seems not to be relevant) and should make sure that high-risk populations (e.g., children with higher body weight, low SES and girls) are reached as target groups. Our results further indicate that prevention of WBI should focus on the enhancement of self-esteem and positive body image. Recently, body positivity is discussed as a promising approach for prevention, including efforts to change appearance ideals and increase acceptance and appreciation of one’s own body [50, 51]. Besides, as depressive problems were also predictive for WBI, focusing on negative affect might also be important. Overall, our prospective study extends the previous cross-sectional research and highlights the importance of intrapersonal variables in the context of WBI.

## Data Availability

The datasets generated and analyzed during the current study are not publicly available, as the participants were not asked for consent concerning publication within repositories, but are available from the corresponding author on reasonable request.

## Abbreviations

BCa 95% CI: 95% bias-corrected and accelerated confidence interval
BMI-SDS: Body mass index standard deviation score
SES: Socioeconomic status
T1 / T2 / T3: Measurement waves 1 / 2 / 3
WBI: Weight bias internalization
WBIS-C: Weight Bias Internalization Scale for children

## References

[1] Puhl RM, King KM (2013). Weight discrimination and bullying. Best Pract Res Clin Endocrinol Metab.

[2] Major B, Tomiyama AJ, Hunger JM, Major B, Dovidio JF, Link BG (2018). The negative and bi-directional effects of weight stigma on health. The Oxford handbook of stigma, discrimination, and health.

[3] Pont SJ, Puhl RM, Cook SR, Slusser W (2017). Stigma experienced by children and adolescents with obesity. Pediatrics.

[4] Di Pasquale R, Celsi L (2017). Stigmatization of overweight and obese peers among children. Front Psychol.

[5] Harter S (2012). The construction of the self: Developmental and sociocultural foundations.

[6] Puhl RM, Latner JD (2007). Stigma, obesity, and the health of the nation’s children. Psychol Bull.

[7] Corrigan PW, Rao D (2012). On the self-stigma of mental illness: stages, disclosure, and strategies for change. Can J Psychiatry.

[8] Durso LE, Latner JD (2008). Understanding self-directed stigma: Development of the Weight Bias Internalization Scale. Obesity.

[9] Papadopoulos S, Brennan L (2015). Correlates of weight stigma in adults with overweight and obesity: A systematic literature review. Obesity.

[10] Pearl RL, Puhl RM (2016). The distinct effects of internalizing weight bias: An experimental study. Body Image.

[11] Zuba A, Warschburger P (2017). The role of weight teasing and weight bias internalization in psychological functioning: A prospective study among school-aged children. Eur Child Adolesc Psychiatry.

[12] Maughan B, Collishaw S, Thapar A, Taylor E, Leckman JF, Snowling MJ, Scott S (2015). Development and psychopathology: a life course perspective. Rutter’s child and adolescent psychiatry.

[13] Corrigan PW, Watson AC (2002). The Paradox of Self-Stigma and Mental Illness. Clin Psychol Sci Prac.

[14] Ratcliffe D, Ellison N (2015). Obesity and internalized weight stigma: a formulation model for an emerging psychological problem. Behav Cogn Psychother.

[15] Puhl RM, Himmelstein MS (2018). Weight bias internalization among adolescents seeking weight loss: Implications for eating behaviors and parental communication. Front Psychol.

[16] Zuba A, Warschburger P (2018). Weight bias internalization across weight categories among school-aged children. Validation of the Weight Bias Internalization Scale for Children. Body Image.

[17] Chan KL, Lee CSC, Cheng CM, Hui LY, So WT, Yu TS, Lin C-Y (2019). Investigating the relationship between weight-related self-stigma and mental health for overweight/obese children in Hong Kong. J Nerv Ment Dis.

[18] Ciupitu-Plath C, Wiegand S, Babitsch B (2018). The Weight Bias Internalization Scale for Youth: Validation of a specific tool for assessing internalized weight bias among treatment-seeking German adolescents with overweight. J Pediatr Psychol.

[19] Roberto CA, Sysko R, Bush J, Pearl RL, Puhl RM, Schvey NA, Dovidio JF (2012). Clinical correlates of the weight bias internalization scale in a sample of obese adolescents seeking bariatric surgery. Obesity.

[20] Maïano C, Aimé A, Lepage G, Morin AJS (2019). Psychometric properties of the Weight Self-Stigma Questionnaire (WSSQ) among a sample of overweight/obese French-speaking adolescents. Eat Weight Disord.

[21] Pakpour AH, Tsai M-C, Lin Y-C, Strong C, Latner JD, Fung XCC (2019). Psychometric properties and measurement invariance of the Weight Self-Stigma Questionnaire and Weight Bias Internalization Scale in children and adolescents. Int J Clin Health Psychol.

[22] Wong PC, Hsieh Y-P, Ng HH, Kong SF, Chan KL, Au TYA (2019). Investigating the self-stigma and quality of life for overweight/obese children in Hong Kong: a preliminary study. Child Ind Res.

[23] Puhl RM, Himmelstein MS, Quinn DM (2018). Internalizing weight stigma: Prevalence and sociodemographic considerations in US adults. Obesity.

[24] Pearl RL, Himmelstein MS, Puhl RM, Wadden TA, Wojtanowski AC, Foster GD (2019). Weight bias internalization in a commercial weight management sample: prevalence and correlates. Obes Sci Pract.

[25] Kromeyer-Hauschild K, Wabitsch M, Kunze K, Geller F, Hesse V, von Hippel A (2001). Perzentile für den Body-Mass-Index für das Kindes- und Jugendalter unter Heranziehung verschiedener deutscher Stichproben [Percentiles of body mass index in children and adolescents evaluated from different regional German studies]. Monatsschr Kinderheilkd.

[26] Thompson JK, Cattarin J, Fowler B, Fisher E (1995). The Perception of Teasing Scale (POTS): A revision and extension of the Physical Appearance Related Teasing Scale (PARTS). J Pers Assess.

[27] Bullinger M, Brütt AL, Erhart M, Ravens-Sieberer U (2008). Psychometric properties of the KINDL-R questionnaire: results of the BELLA study. Eur Child Adolesc Psychiatry.

[28] Döpfner M, Lehmkuhl G, DISYPS-KJ (2000). Diagnostik-System für psychische Störungen im Kindes- und Jugendalter nach ICD-10 und DSM-IV [DISYPS-KJ: Diagnostic system for mental disorders in childhood and adolescence].

[29] Field AP (2013). Discovering statistics using IBM SPSS statistics: And sex and drugs and rock’n’roll.

[30] Little RJA, Rubin DB (2002). Statistical analysis with missing data.

[31] Enders CK (2010). Applied missing data analysis.

[32] Cohen J (1988). Statistical power analysis for the behavioral sciences.

[33] Pearl RL, White MA, Grilo CM (2014). Overvaluation of shape and weight as a mediator between self-esteem and weight bias internalization among patients with binge eating disorder. Eat Behav.

[34] Hayes AF (2018). Introduction to mediation, moderation, and conditional process analysis: A regression-based approach.

[35] Boswell RG, White MA (2015). Gender differences in weight bias internalisation and eating pathology in overweight individuals. Adv Eat Disord.

[36] Weinberger N-A, Kersting A, Riedel-Heller SG, Luck-Sikorski C (2016). Body dissatisfaction in individuals with obesity compared to normal-weight individuals: A systematic review and meta-analysis. Obes Facts.

[37] Trzesniewski KH, Donnellan MB, Robins RW (2003). Stability of self-esteem across the life span. J Pers Soc Psychol.

[38] Furnham A, Badmin N, Sneade I (2002). Body image dissatisfaction: gender differences in eating attitudes, self-esteem, and reasons for exercise. J Psychol.

[39] van den Berg PA, Mond J, Eisenberg M, Ackard D, Neumark-Sztainer D (2010). The link between body dissatisfaction and self-esteem in adolescents: similarities across gender, age, weight status, race/ethnicity, and socioeconomic status. J Adolesc Health.

[40] Kline P (1999). The handbook of psychological testing.

[41] Nunnally JC (1978). Psychometric theory.

[42] Pedhazur EJ, Schmelkin LP (2013). Measurement, Design, and Analysis: An Integrated Approach.

[43] Bühner M (2011). Einführung in die Test- und Fragebogenkonstruktion [Introduction to test and questionnaire construction].

[44] Rasmussen JL (1989). Analysis of Likert-scale data: A reinterpretation of Gregoire and Driver. Psychol Bull.

[45] Zumbo BD, Zimmerman DW (1993). Is the selection of statistical methods governed by level of measurement?. Can Psychol.

[46] Jamieson S (2004). Likert scales: how to (ab)use them. Med Educ.

[47] Baker BO, Hardyck CD, Petrinovich LF (1966). Weak measurements vs. strong statistics: An empirical critique of S. S. Stevens’ proscriptions on statistics. Educ Psychol Meas.

[48] Hilbert A, Braehler E, Haeuser W, Zenger M (2014). Weight bias internalization, core self-evaluation, and health in overweight and obese persons. Obesity.

[49] Lee M, Ata RN, Brannick MT (2014). Malleability of weight-biased attitudes and beliefs: A meta-analysis of weight bias reduction interventions. Body Image.

[50] Watkins PL, Clifford D, Souza B, Daniels EA, Gillen MM, Markey CH (2018). The Health At Every Size® paradigm: Promoting body positivity for all bodies. Body positive: Understanding and improving body image in science and practice.

[51] Cohen R, Irwin L, Newton-John T, Slater A. #bodypositivity. A content analysis of body positive accounts on Instagram. Body Image. 2019;29:47–57. doi:10.1016/j.bodyim.2019.02.007. 10.1016/j.bodyim.2019.02.007 30831334

